# Cooperative foraging during larval stage affects fitness in *Drosophila*

**DOI:** 10.1007/s00359-020-01434-6

**Published:** 2020-07-04

**Authors:** Mark Dombrovski, Rives Kuhar, Alexandra Mitchell, Hunter Shelton, Barry Condron

**Affiliations:** 1 Department of Biology, University of Virginia, Charlottesville, VA 22901 USA; 2 Present Address: Department of Biological Chemistry, HHMI, David Geffen School of Medicine, University of California, Los Angeles, Los Angeles, CA 90095 USA

**Keywords:** *Drosophila*, Foraging, Cooperation, Social behavior, Fitness

## Abstract

Cooperative behavior can confer advantages to animals. This is especially true for cooperative foraging which provides fitness benefits through more efficient acquisition and consumption of food. While examples of group foraging have been widely described, the principles governing formation of such aggregations and rules that determine group membership remain poorly understood. Here, we take advantage of an experimental model system featuring cooperative foraging behavior in *Drosophila*. Under crowded conditions, fly larvae form coordinated digging groups (clusters), where individuals are linked together by sensory cues and group membership requires prior experience. However, fitness benefits of *Drosophila* larval clustering remain unknown. We demonstrate that animals raised in crowded conditions on food partially processed by other larvae experience a developmental delay presumably due to the decreased nutritional value of the substrate. Intriguingly, same conditions promote the formation of cooperative foraging clusters which further extends larval stage compared to non-clustering animals. Remarkably, this developmental retardation also results in a relative increase in wing size, serving an indicator of adult fitness. Thus, we find that the clustering-induced developmental delay is accompanied by fitness benefits. Therefore, cooperative foraging, while delaying development, may have evolved to give *Drosophila* larvae benefits when presented with competition for limited food resources.

**Electronic supplementary material:**

The online version of this article (10.1007/s00359-020-01434-6) contains supplementary material, which is available to authorized users.

## Introduction

Group foraging is a major component of cooperative animal behavior (Allee 1927). It can be defined as inter- and intraspecific cooperation in search, acquisition, defense and consumption of common food resources, and can provide benefits in survival and reproduction for a variety of animals (Sumpter 2005; Giraldeau and Caraco 2018). Participation in a cooperative feeding group can provide a significant enhancement in average feeding efficiency for two reasons: (1) increased food processing efficiency resulting in less investment for higher nutritional return (Valone 1989; Cash et al. 1993; Pöysä 1994; Tripet et al. 2009) and (2) a potential to sequester a common food resource from competing species or different populations of the same species (Foster 1985; Dubois 2003; Tania 2012). In addition, aggregation can also lead to a decreased risk of predation (Turchin and Kareiva 1989; Rohlfs and Hoffmeister 2004). All of these factors increase an individual’s chances of survival and reproductive success which serve as the main measures of fitness (Clark and Mangel 1986). Importantly, benefits of cooperative foraging often take effect under certain conditions when availability and distribution of food resources determine the advantage of cooperation (Monaghan and Metcalfe 1985; Scheel and Packer 1991; Eklöv 1992; Amor et al. 2010). This may serve an example of Allee effect (Courchamp et al. 1999) in context of cooperative feeding, where an individual’s fitness gains correlate with group size and density only to a certain limit, beyond which acquired benefits get leveled and are negatively outweighed by emerging complex non-trophic factors of group membership, such as intra-group competition (Clark 1987; Cash et al. 1993; Rivers et al. 2011).

Examples of cooperative foraging providing fitness benefits have been documented among a broad range of animal taxa. Group hunting strategies that increase success rate of prey localization and acquisition were described in carnivorous mammals (Clark 1987), birds (Hector 1986), and fish (Eklöv 1992), all of which predominantly utilize active coordinated hunting tactics. Herbivores widely engage in cooperative foraging (Monaghan and Metcalfe 1985; Foster 1985; Pöysä 1994), which helps in interspecific competition for a limited food resource. Invertebrates and, in particular, arthropods also display a variety of cooperative foraging behaviors associated with fitness gains including active hunters (Schneider and Bilde 2008) and scavengers (Amor et al. 2010) that congregate in groups. Phytophagous insects (Tsubaki and Shiotsu 1982; Cocroft 2005) display increased survival and reproduction through more economical consumption of food resources when working in groups. Blood-sucking insects (Tripet et al. 2009), reduce expenditure of resources during cooperative blood feeding and this results in increased fecundity. Numerous examples of interspecific aggregations are described as well (Boulay et al. 2019). Many studies focus on insect larvae in which food consumption is a top priority (Fitzgerald and Peterson 1988), and animals are, therefore, very sensitive to trophic advantages of group membership. Indeed, highly efficient foraging aggregations that enhance survival rates compared to solitary foragers are documented in sawfly larvae (Ghent 1960), various species of caterpillars (Clark and Faeth 1997), where animals reared in larger groups displayed higher developmental rates and increased survival. This is also true for corpse-devouring necrophagous flies (Scanvion et al. 2018; Aubernon et al. 2019). Importantly, factors that provide trophic benefits can serve as tradeoffs in case of severe overcrowding (e.g., overly elevated temperature and proteotoxic stress caused by excessive tissue digestion, exemplified by Rivers et al. 2011), implying a complex non-linear pattern of relationship between group size (Cash et al. 1993) and composition (Trowbridge 1991), food availability and distribution (Monaghan and Metcalfe 1985), presence or absence of predators (Turchin and Kareiva 1989) and individuals’ investment into cooperative efforts (Valone 1989; Courchamp 1999; Giraldeau and Caraco 2018; Lindstedt et al 2018). In this regard, using a laboratory model system might provide the right tools and metrics to begin dissecting out various complex parameters governing collective foraging behavior.

To address these questions, we make use of a novel experimental model system featuring cooperative foraging behavior in larval *Drosophila melanogaster*. Interestingly, although behavioral and developmental aspects of larval solitary foraging behavior were addressed a long time ago (Sokolowski 1982; Godoy-Herrera 1977, 1986; Wu et al. 2003; Kim et al. 2017), mechanistic and neuroethological features of cooperative foraging in larval *Drosophila* have only recently been characterized (Durisko et al. 2014; Dombrovski et al. 2017, 2019; Khodaei and Long 2019). Feeding clusters form in semi-liquid food, comprised of 10–200 animals and share a unique set of characteristics that make it an attractive model for studying collective social behavior. In particular, clustering larvae engage in synchronous reciprocating digging, where each group member utilizes visual cues to coordinate movements with immediate neighbors (Dombrovski et al. 2017). Intriguingly, cluster membership and ability to efficiently engage in visually guided cooperation require prior visual and social experience during a critical period in development (Slepian et al. 2015; Dombrovski et al. 2017). In addition, emergence of clustering is associated with functional changes of larval visual circuit (Dombrovski et al. 2019). This raises the question as to the function of this behavior and its emergence in evolution. The main goal of our study was to find out whether engagement in larval social foraging clusters is associated with any fitness benefits at later developmental stages.

## Materials and methods

### Fly stocks

Wild-type Canton S (CS) flies were donated by Ed Lewis (Caltech), blind GMR-hid^G1^ strain was obtained from Bloomington Stock Center (#5771), and w^−^; Sp/CyO; TM2/TM6B-Tb flies were kindly provided by Dr. Susan Doyle, University of Virginia.

### Fly stock maintenance, egg collection and larval rearing

All *Drosophila melanogaster* stocks were raised in food vials (unless further specified in experimental details) containing standard Caltech food mixture (1000-ml molasses, 14000-ml H_2_O, 148-g agar, 1000-ml corn meal, 412-g Baker’s Yeast, 225-ml Tegosept, 80-ml propionic acid) at 22 °C, 30% humidity under standard 12/12 h light–dark cycle (unless further specified in experiments involving dark rearing) at ~ 1000 lx light intensity. For egg production, ~ 50 adult flies 3–4 days old were transferred into egg cups and kept in the same conditions. Eggs were laid on 35-mm Petri dishes (egg plates) containing standard agar-molasses food and yeast. For collection of larvae at designated developmental stages, egg plates were changed every 6 h and subsequently kept (unless further specified) in the same light/temperature/humidity conditions. Depending on the experimental purposes, larvae were collected from egg plates in the middle of L2 stage (60 h after egg laying, abbreviated further as hAEL), or middle of L3F2 stage (110 hAEL).

### Preparation of vials with “pre-processed” food and larval transplantations

**Fig. 1:**
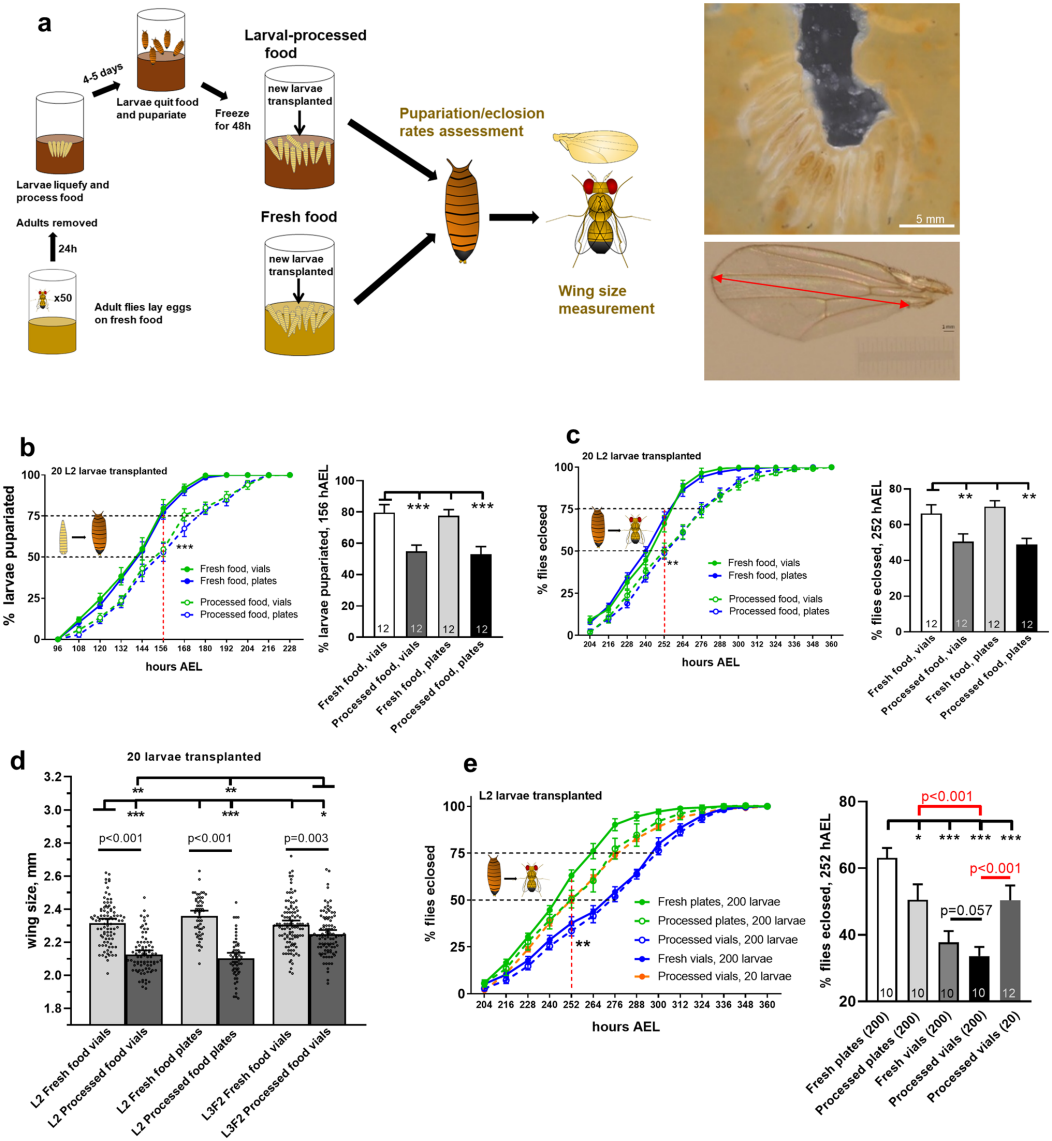
Processed food delays larval development and decreases animal size. **a** Schematic view of experimental procedures. To produce processed food, ~ 50 adult flies were kept in fresh food vials for 24 h and then removed, allowing a sufficient number of larvae to hatch and liquefy food within the next 4–5 days. After all larvae pupariated, vials were frozen for 48 h and cleaned. Newly collected larvae at a designated developmental stage were transplanted into defrosted vials with processed food in parallel with fresh food vials. Eclosion and pupariation rates were measured followed by assessment of adult female fly wing size to examine the effect of processed food on animal growth and development. **b** and **c** Processed food causes a significant developmental retardation. 20 L2 wild-type larvae were transplanted in vials or plates with fresh or processed food and subsequently evaluated for developmental rates. Rearing in processed food results in a consistent ~ 16–24-h delay in both pupariation **b** and eclosion **c**. No difference was seen between plates and vials. Percentage of larvae pupariated/eclosed was measured every 12 h starting at 96 and 192 h AEL, respectively. Here and further on, 156-h AEL and 252-h AEL checkpoints (indicated by dashed red lines) were compared for pupariation **b** and eclosion **c**, respectively, with data represented in bar graphs. **d** Processed food decreases wing size in adult animals. 20 L2 wild-type larvae were transplanted into plates or vials with fresh or processed food with subsequent evaluation of adult fly wing size. In addition, effect of late transplantation (L3F2 stage) was examined and compared between fresh and processed food vials. A significant decrease in adult wing size was observed in L2 transplants raised in processed food, regardless of rearing in plates or vials. A smaller reduction in adult wing size was seen in L3F2 transplants raised in processed food. **e** Crowded conditions exacerbate developmental retardation. Pupariation rates were assessed in 20 and 200 L2 wild-type larvae transplanted into plates or vials with fresh or processed food. No difference was found between fresh and processed food vials. 200 larvae raised in processed vials showed the biggest delay in pupariation, being significantly different from both 20 animals in processed food vials and 200 animals in processed food plates (right panel, highlighted in red)

Techniques of “pre-processed” vial production and transplantation of 200 L2 (60 hAEL) larvae were adapted from our previous studies (Dombrovski et al. 2017, 2019 and Fig. 1a). About 50 adult flies, 3–4 days old, were kept in vials with fresh food for 24 h and subsequently removed; vials remained at standard conditions for 4–5 days allowing larvae to process food and pupariate. Then, vials were frozen at  − 20 °C for 48 h, defrosted and cleaned off pupae before new larvae were transplanted into processed food. This approach was developed to minimize the variance of “pre-processed’ food resources along with the absence of any unwanted animals and immediate exposure of transplanted larvae to designated food conditions.

### Cluster frequency measurements

For cluster frequency measurements, vials with previously transferred 200 L2/L3F2 larvae of a designated genotype/condition raised in normal conditions were recorded for 5 consecutive days (24 h non-stop) starting immediately after transplantation. Videos were recorded on an iPhone 5 at full resolution and 1 frame/60″ using “Lapseit” software for iOS and subsequently analyzed using iMovie and ImageJ (32-bit version for Windows). Percentage of frames with a clearly observed larval cluster (defined as a group of 5 or more larvae aligned and oriented vertically and buried into the food for more than ¾ of the body length) was calculated for each 12-h light period during 3 consecutive days of recordings for each individual vial (days 3–5 after transplantation for L2 larvae and days 1–3 after transplantation for L3F2 larvae) of a designated genotype/condition (each of these measurements represents a single data point on the corresponding graphs). Values were subsequently averaged and represent mean values for each genotype/condition.

### Pupariation, eclosion and survival rates measurements

Newly formed pupae were counted on each food vial/plate of a designated genotype/condition twice a day (equal 12-h periods and highlighted with a marker to avoid repeat counting). Eclosed flies were counted (for survival rates evaluation) and collected using CO2 anesthesia twice a day (equal 12-h periods), and females were subsequently frozen at  − 20 °C in 1.5-mL plastic tubes for subsequent wing size measurements. For pupariation and eclosion measurements, percentage of animals that reached a designated developmental stage was calculated relative to the total number of pupariated/eclosed animals counted by the final day of observations (not the total number of originally transplanted larvae). Survival values were estimated as ratios of eclosed flies to the total number of originally transplanted larvae. In the case of mixed populations, only wild-type CS flies were counted.

### Dark-rearing experiments

In all experiments involving light deprivation, dark-reared larvae were kept in a completely dark room for a designated time period in a food plate/vial and were additionally covered with a layer of aluminum foil. Dark rearing began immediately after larval transplantation at a designated stage and until the eclosion of all adult flies. Daily eclosion and pupariation rate measurements were performed in a room with dim red lights for each vial individually to minimize the time of possible light exposure.

### Wing size measurements

Wing size (which serves as an estimate of body size) of previously collected and frozen females was measured using a technique adapted from Gilchrist and Partridge (1999) (distance from the base of the alula to the distal end of the third longitudinal vein, see Fig. 1a). A single wing from each animal was removed and mounted on a slide; each slide represented 15–20 wings derived from animals from a single food plate/vial, yielding in 4–6 slides per genotype/condition and an individual wing measurement represented a single data point on the corresponding graphs. High-quality images of the slide were taken with a camera mounted on a tripod for subsequent wing size assessment using ImageJ (see below). Nikon D3100 CMOS camera with 50-mm lens and fitted with a Raynox Macroscopic 4 × lens was used. Values were then averaged to give an estimate of the wing size for a designated genotype/condition.

### Statistical analysis

Unless otherwise stated, all data are presented as mean values and error bars represent 95% confidence intervals. Statistical significance was calculated by one-way ANOVA using Tukey’s method. When comparing two groups of normally distributed data, Student’s two-tailed unpaired T-test was used. *p < 0.05; **p < 0.01; ***p < 0.001. Linear regression analysis was used for Fig. 3c, d and S2c, d. All analyses were performed using the GraphPad Prism 8 statistical software for Windows.

## Results

### Processed food delays development and reduces size in *Drosophila*

Processed food and crowded conditions increase the duration of larval development (Dombrovski et al. 2017). Here, we further investigated the separate contribution of each of these factors in more detail. Preparation of vials with processed food followed by larval transplantations was adapted from previous studies (Dombrovski et al. 2017, 2019, Fig. 1a). In our first experiment (Fig. 1b), we compared the effects of fresh versus processed food on larval pupariation and eclosion rates. To exclude the role of cooperative feeding, we used a low-population density paradigm and transplanted 20 L2 wild-type larvae (previously raised in normal conditions on fresh food) into plates and vials with processed food. We found that both pupariation (Fig. 1b) and eclosion (Fig. 1c) were significantly delayed in animals raised on processed food, but no difference was observed between rearing in plates and vials. Importantly, no effect on survival was found (Fig. S1a, left) and blind GMR-hid larvae displayed a similar eclosion delay in processed food (Fig. S1b). In summary, processed food yielded in a ~ 16-h delay in pupariation and in eclosion in which a sub-cooperative number (20) of larvae were transplanted at L2 stage.

It has been reported that insufficient nutrition during larval stage reduces size in adult flies (Colombani et al 2003; Mirth and Riddiford 2007; Lavalle et al. 2008). Therefore, we next examined whether an observed developmental retardation was associated with size deficits. For this, we measured wing size in newly eclosed female flies, which serves a good estimate of general body size and weight (Taylor et al. 2008; Tang et al. 2011). We found that, regardless of rearing on plates or in vials, animals reared on fresh food had significantly bigger wings (Fig. 1d), suggesting a negative impact of processed food on larval growth. In addition, we performed L3F2 larval stage transplantations into processed food vials and plates to see if decreased time spent in an adverse nutritional environment would fully or partially rescue size deficits. We found that wing size in adults derived from L3F2 transplants raised on processed food was significantly smaller compared to fresh food-reared larvae (Fig. 1d). However, the effect of food on L3F2 transplants was less pronounced than in case of L2 transplants, suggesting that time spent in processed food during larval stage negatively correlates with adult size. Alternatively, these results could also be explained by the fact that transplantation occurred after reaching critical weight (Mirth et al. 2005; Mirth and Riddiford 2007, see “Discussion” for details). Survival rates were unchanged among all experimental paradigms (Fig. S1a).

We next wondered how high population density changed the way processed food affects developmental timing especially accounting for the appearance of cooperative foraging. For that, we transplanted either 20 or 200 wild-type L2 larvae in vials and plates (cooperative foraging technically possible in the former but not latter case) with either fresh or processed food and compared their pupariation and eclosion rates (Fig. 1e and S1c). There was no difference in developmental rates between fresh and processed vials (Fig. 1e and S1c), which can be explained by very fast food processing by 200 animals. However, larvae reared in high density in vials with processed food displayed dramatically delayed pupariation and eclosion compared not only to fresh food plates but also to processed food plates and processed food vials with low-density conditions (Fig. 1e and S1c). For control, GMR-hid larvae unable to perform efficient group foraging (Dombrovski et al. 2017, 2019) were exposed to similar experimental conditions (Fig. S1b, left and middle panels). As opposed to wild-type animals, blind counterparts displayed no significant differences in developmental timing between high (200) and low (20) population density experimental paradigms. This evidence pointed to the role of cooperative foraging clusters in delaying larval development and this assumption was further examined.

### Cooperative foraging further delays larval development in processed food at high population density

**Fig. 2:**
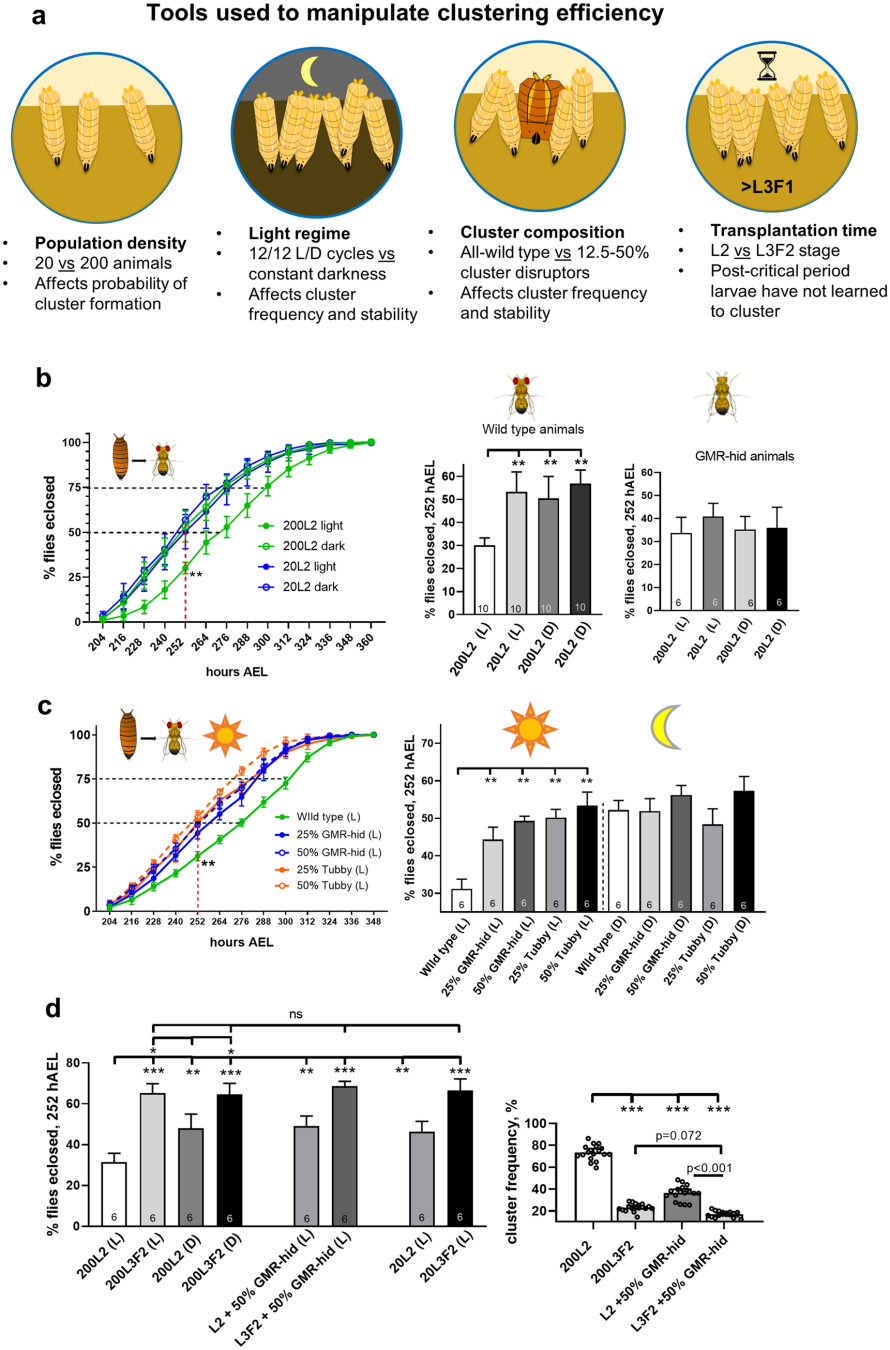
Cooperative foraging further delays larval development in processed food. **a** Schematic view of approaches further used (individually or in various combinations) to affect clustering efficiency in wild-type animals. **b** Light regime affects larval developmental rates, but only in conditions that otherwise promote clustering. Eclosion rates were compared between 20- and 200 L2-transplanted wild-type larvae reared in normal light conditions (L) and in the darkness (D). Eclosion delay was significantly reduced in dark-reared 200 L2 larvae compared to animals raised in normal light conditions. However, no significant difference was found in case of 20 L2 transplants (left and middle panels). In addition, no difference in developmental timing was seen among blind GMR-hid larvae exposed to the same experimental conditions (right panel). **c** Addition of cluster disruptors reduces larval developmental delay, but only in conditions that otherwise promote clustering. Eclosion rates were compared between 200 L2 transplant groups containing 100% wild-type larvae, 75% wild-type + 25% GMR-hid or Tubby and 50% wild-type + 50% GMR-hid or Tubby larvae. Same experiments were performed in the darkness. For experiments performed in normal light conditions, addition of blind or Tubby larvae resulted in a significantly decreased delay in eclosion compared to all-wild-type groups. This was coupled with a corresponding decrease in clustering frequency (see figure S2c, left panel). At the same time, no difference in eclosion rates was found between all-wild-type and mixed groups for dark-reared animals (see figure S1e). **d** Transplantation after critical period for clustering reduces developmental delay. Eclosion rates were compared between wild-type larvae transplanted at L2 and L3F2 stages, including comparison between 20 and 200 animals, normal light conditions (L) and dark rearing (D) as well as all-wild-type populations and mixed groups containing 50% blind GMR-hid larvae. Regardless of any manipulations, L3F2 transplants displayed a significantly reduced delay in eclosion compared to 200 L2 larvae in normal light conditions, 200 L2s in the darkness and 20 L2s as well as 200 L2s in a mixed group (left panel). At the same time, L3F2 transplants showed significantly reduced clustering frequency compared to control wild-type 200 L2 and even mixed group 200 L2 larvae (right panel)

To examine the role of cooperative foraging in larval development, we took advantage of approaches shown in Fig. 2a. In particular, the emergence of clustering requires certain population density with optimal values around 200 animals per food vial (Dombrovski et al. 2017). We previously demonstrated that wild-type animals display dramatically reduced clustering when either deprived from light during specific periods of development (Dombrovski et al. 2019) or immediately after being placed in the darkness (Dombrovski et al. 2017). Here, we compared developmental rates in wild-type larvae reared in normal conditions and in the darkness, and we observed a significant delay in pupariation and eclosion times (Fig. 2b and S1d) in normally reared animals compared to dark-reared counterparts. However, this effect was notable only in cases of high population density that promotes clustering and no difference was found in cases of 20 animals (Fig. 2b and S1d). Moreover, no difference in developmental timing was seen between normal- and dark-reared GMR-hid larvae that cannot form clusters (Fig. 2b, right panel and S1b, left and middle panels). Animal survival rates were not affected by dark rearing (Fig. S1b, S1d). This has further strengthened our notion of social clustering being an important factor responsible for an increased developmental delay.

To provide more evidence in support of this hypothesis, we used cluster “disruptors” as another tool to manipulate clustering frequency (Fig. 2a), as our previous study indicates that introduction of non-clustering larvae into wild-type foraging groups decreases cluster lifetime (Dombrovski et al. 2017). For this experiment, we compared developmental timing in all-wild-type 200 L2 larval groups and mixed groups (Fig. 2c) containing 25 and 50% GMR-hid or Tubby (Tb) larvae; both negatively interfering with clustering through their inability to either integrate into or efficiently cooperate within a cooperative group, as shown by Dombrovski et al. (2017). Vials were constantly recorded and cluster frequency in each vial was further assessed (Fig. S2c, left). The same experimental conditions were reproduced in dark-reared larvae. We saw that the length of a delay in eclosion highly correlated with clustering frequency; it was most notable in all-wild-type group and significantly decreased in a stepwise manner in 25% and 50% GMR-hid/Tb groups, similar to a decrease in clustering frequency observed among the corresponding groups (Fig. S2c). Most importantly, no effect of group composition on developmental timing was seen in animals reared in the darkness (Fig. S1e, left), suggesting that clustering could be a decisive factor. Survival rates were unchanged between light/dark conditions and group compositions (Fig. S1e, right).

Earlier studies also indicate that to cluster, larvae must pass through a visual critical period early in the third instar (Dombrovski et al. 2017, 2019), and animals transplanted into vials of processed food after this critical period show greatly reduced clustering. Therefore, we tested the effects of reducing clustering by post-visual critical period transplantation. We also reproduced the same experiment in the darkness, with 20 larvae and with a mixed group containing 50% GMR-hid larvae. We found that in standard high-density conditions in light, L3F2-transplanted larvae displayed a significantly reduced delay in eclosion compared to 200 L2 and even 20 L2 transplants (Fig. 2d and S2a). Moreover, neither dark rearing nor adding blind larvae and reducing animal density yielded a significant change in eclosion rates of L3F2 transplants*. *Importantly, clustering frequency was significantly reduced in L3F2 transplants (Fig. 2d, right) and was not affected by adding cluster disruptors (GMR-hid animals). These results implied that, since cooperative foraging was eventually absent in late-transplanted animals (Dombrovski et al. 2017), none of the factors reducing clustering efficiency affected their development, as opposed to L2-transplanted larvae being very sensitive to each factor (Fig. 2d and S2a). However, other interpretations of these results are possible. The fact that L3F2-transplanted blind GMR-hid larvae also showed a reduced developmental delay compared to L2 transplants (Fig. S2b) strongly suggested that the observed phenotype could also result from post-critical weight transplantation, thus making the effect of reduced clustering negligible. Taken together, our experiments strongly suggested that engagement of larvae in social foraging groups was strongly associated with subsequent developmental retardation that significantly exceeded a delay in metamorphosis caused by processed food. Identifying consequences associated with this delay was a subject of subsequent experiments.

### Cooperative foraging during larval stage rescues size deficits caused by processed food in adult *Drosophila*

**Fig. 3:**
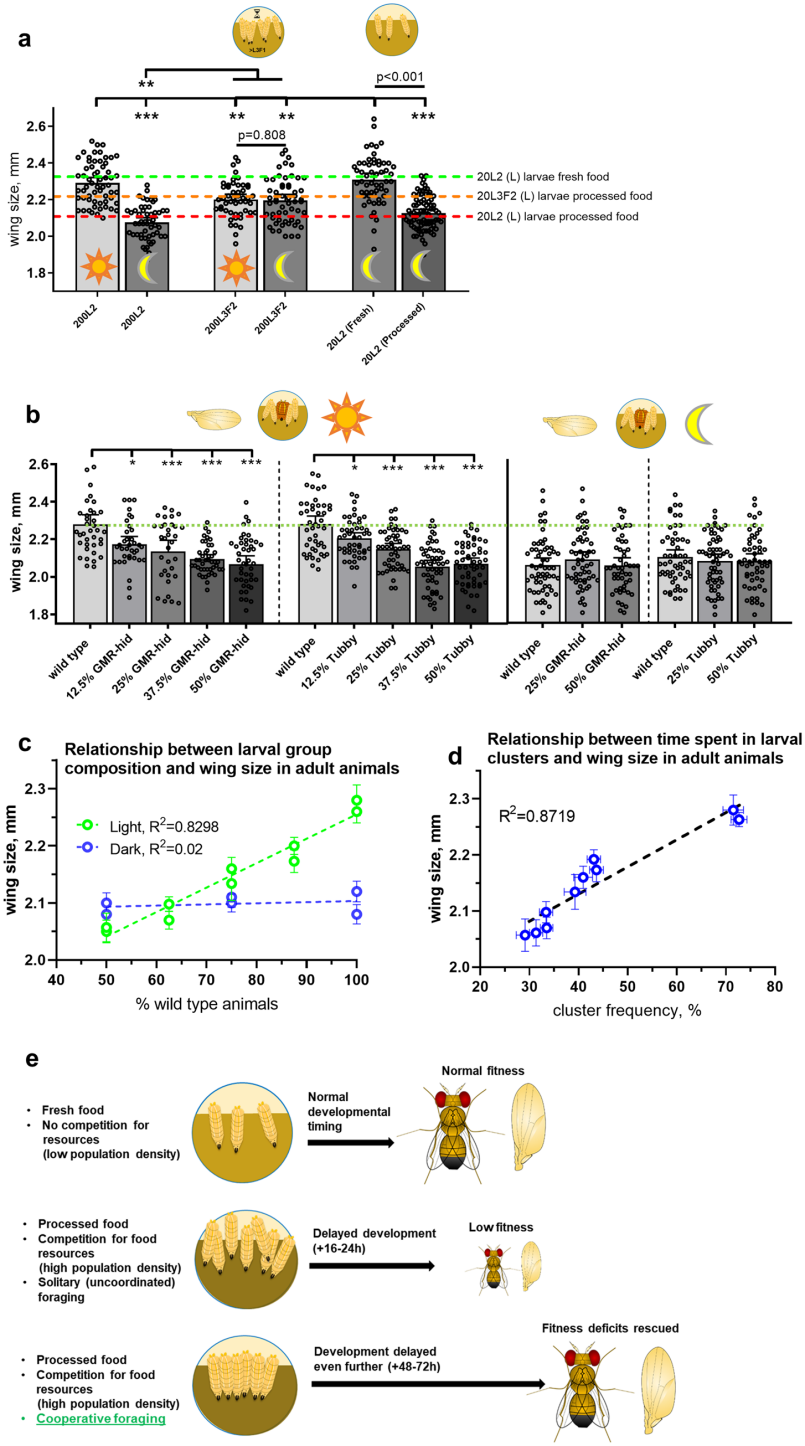
Clustering rescues fitness deficits caused by processed food. **a** Conditions that promote clustering also positively affect wing size. Wings size was compared between wild-type animals derived from light- and dark-reared L2 and L3F2 transplants (experiment shown in Fig. 2d). 200 L2-transplanted larvae reared in normal light conditions (L) give rise to adults that have significantly bigger wings compared to their dark-reared counterparts (D) and 20 L2 larvae (indicated by a red dashed line, data from Fig. 1d) and only slightly smaller compared to 20 L2 animals raised on fresh food, serving as a positive control (indicated by a green dashed line, data from Fig. 1d). In contrast, larvae transplanted at L3F2 stage display no difference in adult wing size between light- and dark-reared animals, as well as 20 L3F2 transplants in processed food (indicated by an orange dashed line, data from Fig. 1d). Nevertheless, L3F2 larvae give rise to adults with smaller wings compared to 200 L2 transplants reared in normal light conditions. As an additional control for non-clustering conditions, wing size was compared between adults derived from 20 L2 larvae transplanted in either fresh or processed food vials (two rightmost bars), with animals derived from fresh food having significantly larger wings. **b** Larval group composition affects adult animals wing size, but only in normal light conditions. Wing size was compared between 200 L2 transplant groups containing 100%, 87.5%, 75%, 62.5% and 50% wild-type larvae reared in normal light conditions with the rest of the group comprising either GMR-hid or Tubby larvae (left panel). For dark-reared animals (right panel), 100%, 75% and 50% wild-type groups were used. A significant difference in wing size was found between all wild-type animals from mixed groups and control 100% wild-type groups raised in normal conditions. On the contrary, no difference in wing size was seen between wild-type animals derived from groups of different composition in dark-reared larvae (wild-type larvae from all groups had reduced wings compared to normal light-reared all-wild-type control group). **c** Relationship between larval group composition and adult animal wing size (data are related to Fig. 3b). Significant positive correlation is seen between percentage of wild-type larvae in a light-reared group and wing size in flies derived from wild-type larvae of the corresponding group. No correlation is observed in case of dark-reared animals. Error bars represent SEM. **d** Relationship between time spent in larval feeding clusters and wing size in adult animals. Data are taken from experiments involving 200 L2 wild-type larvae and mixed groups containing 12.5–50% GMR-hid and Tubby larvae in light (results presented in Fig. 3b, left and middle panels; original clustering frequency data are presented in Fig. S2d, top panel). A significant positive correlation is seen between percentage of time a cluster was observed in a group of a designated composition and wing size in flies derived from wild-type larvae of the corresponding group. Error bars represent SEM. **e** Summary of the main findings described in our study. Adverse nutritional environment coupled with high degree of competition for food resources causes significant developmental retardation in *Drosophila* larvae that is associated with decreased adult fitness. However, same conditions also promote formation of cooperative foraging clusters, membership in which delays larval development even further, but allows to rescue fitness deficits in adult animals

The experiments described above show that clustering further increases developmental time. We next examined how this delay affected adult animal size as measured by wing size. First, we compared wing size in flies emerged from 200 L2-transplanted wild-type larvae raised on processed food and reared in normal light conditions and in the darkness (Fig. 3a), considering that light deprivation prevented animals from clustering. We found that wings of dark-reared animals were significantly smaller compared to the control group (Fig. 3a). Interestingly, a similar magnitude of difference in wing size was previously observed between 20 L2-transplanted wild-type animals raised on fresh and processed food (Fig. 3a, dashed green and red lines, respectively). Thus, wing size was almost indistinguishable between animals raised on fresh food and animals derived from processed food, but only in conditions that promoted clustering (high population density and normal light regime). This suggests that clustering rescued the deficit in animal size caused by processed food. To further elucidate this phenomenon, we looked at how other factors interfering with clustering (Fig. 2a) affected wing size. To account for population density as a factor that affects cooperative foraging, we compared wing size between animals derived from 20 L2 transplants in fresh vs processed food and found that wing size in animals derived from fresh food was significantly larger than in processed food-reared counterparts (Fig. 3a, two rightmost bars). This strengthened a notion that negative effect of processed food on fitness cannot be rescued in the absence of clustering. Next, to account for late post-critical period transplantation that prevents larvae from clustering, we replicated these experiments with 200 L3F2-transplanted larvae reared in light or darkness (Fig. 3a, middle bars). Results intermediate in that wings in flies derived from L3F2 transplants were significantly smaller compared to positive control (20 L2 transplants in light), but they were also notably larger than the negative control (200 L2 in the darkness and 20 L2 in processed food). However, wing size in 200 L3F2-derived animals was almost identical to 20 L3F2 transplants and not affected by light regime (Fig. 3a). This was in line with the data on L3F2 eclosion rates (Fig. 2d and S2a), implying a possible complex interplay between factors mentioned in Fig. 2a and additional aspects such as post-critical weight effects in regulation of developmental timing and determination of adult body size (Mirth et al. 2005; Mirth and Riddiford 2007; Mirth and Shingleton 2012).

To find more reliable correlation between changes in clustering and its effect on animal size, we compared the wing size in wild-type animals reared in clustering conditions but with cooperative behavior reduced in a regulated manner by the addition of cluster disruptors (Fig. 2c and S1e). We added 12.5%–50% of either GMR-hid or Tb larvae to wild-type animals transplanted at L2 stage. Clustering frequency in each group was assessed (as described in Dombrovski et al. 2017), and compared to the same conditions in darkness (Fig. S2d). A clear relationship was seen between group composition and wild-type wing size in light-reared animals (Fig. 3b, left and c): wing size decreased as clustering was suppressed (Fig. S2d). At the same time, we found no difference in wild-type wing size between groups of different composition reared in the darkness (Fig. 3b, right and c). Overall, we were able to trace a strong positive correlation between the time wild-type animals spent in clusters and their resulting adult size (Fig. 3d). In contrast, none of the aforementioned factors affected wing size in blind GMR-hid animals (Fig. S2e); while the overall negative impact of processed food on wing size was still present, which is consistent with the notion that blind animals are unable to use the benefits of cooperative foraging due to incapability to form efficient feeding clusters.

## Discussion

Our study tests the idea that cooperative foraging among fruit fly larvae has a fitness benefit that is measured as body size and developmental time. Overall, we find mixed results in that the duration of larval stage is increased but despite crowding, eclosed adults have normal size when raised in processed food, as opposed to their solitary digging counterparts that display lower fitness in similar conditions (crowding and processed food). Therefore, our study indicates that cooperative foraging in fruit fly larvae can be beneficial in some but not all conditions (Fig. 3e). This result is especially intriguing considering that crowded conditions among *Drosophila* larvae are generally associated with reduced fitness as measured by developmental time, adult survival, body size, fecundity and life span (Lewontin 1955; Miller and Thomas 1958; Horváth et al. 2016), although the exact opposite effects on longevity have been documented as well (Sorensen et al. 2001). This highlights that clustering represents a very special type of larval aggregation largely relying on social interactions, which makes it significantly different from non-cooperative crowding. At the same time, specific mechanisms underlying clustering-associated fitness gains remain to be determined.

Processed food that has been predigested by larvae in our experimental paradigm slows developmental rates (Fig. 1 and S1). However, exact causes and mechanisms of this effect are not fully clear. In the simplest interpretation, processed food has a lower nutritional value or at least an altered ratio of key macronutrients. According to a conventional notion, larval growth, developmental time and final size determination in insects including *Drosophila* are governed by insulin-like hormones and TOR signaling in prothoracic gland (that is highly sensitive to nutritional status), as well as antagonistic actions and complex interplay of ecdysone and juvenile hormone (Colombani et al 2003, 2012; Layalle et al. 2008; Mirth and Shingleton 2012). In this context, malnutrition can have a different impact on larval fate depending on whether it affects an animal before or after reaching critical weight, a key parameter that determines the readiness of the larvae to undergo metamorphosis and triggers the corresponding hormonal signals (Mirth and Shingleton 2012). If occurring before that checkpoint in the middle of L3F1 stage, malnutrition only delays metamorphosis, but does not affect adult fly body size. Conversely, post-critical period starvation later in development has no influence on developmental rates, but dramatically reduces body weight and size (Mirth and Riddiford 2007; Mirth and Shingleton 2012). Interestingly, we observe both effects in solitary feeding animals (in both low- and high population densities), suggesting that processed food provides less nutrients, but not to an extent that would prevent larvae from reaching a minimum viable weight (Mirth and Riddiford 2007). In contrast, adult flies derived from clustering larvae lack size deficits, but display an even longer developmental delay. It implies that once animals engage in cooperative foraging groups, the efficiency of their feeding increases leading to a rescue in body size deficits. This idea is strengthened by our results that showed larvae transplanted into vials at L3F2 stage do not delay metamorphosis (explained by post-critical weight transplantation) but still have reduced body size because they cannot cluster to feed more efficiently (transplantation occurs after critical period for clustering initiation, as shown by Dombrovski et al. 2017). Thus, cooperative foraging can be regarded as an evolutionary adaptation that outweighs malnutrition at the cost of developmental retardation. At the same time, specific mechanisms responsible for an additional delay in metamorphosis observed among clustering larvae remain unclear and are subject for future investigation.

A question arising from previous observations is how clustering enhances the efficiency of food consumption and whether that is an only factor that confers fitness advantages to social foragers. We previously demonstrated that clustering larvae can take advantage of more efficient burrowing and reach deeper layers of food compared to both solitary digging and non-clustering blind or socially naive animals raised in similar crowded conditions with limited food resources (Dombrovski et al. 2017). This could also decrease the probability of predation and infection by parasitoid wasps in natural conditions (Carton et al. 1985). Clustering was shown to speed up the process of media liquefaction in vials (Dombrovski et al. 2017) that could in turn facilitate and speed up food ingestion. A more complex explanation features a phenomenon of communal exodigestion that is mostly observed among various fly larvae feeding on flesh and other high-protein substrates (Scanvion et al. 2018) but was also documented in *Drosophila* (Gregg et al. 1990). Larvae are able to secrete a variety of enzymes that digest external polymers (amylose, cellulose and even chitin), therefore reducing energy expenditure per individual animal required to process and ingest a food source. Future studies are required to test this hypothesis. Furthermore, other factors (both in combination and separately from reduced nutritional value) related to processed food might as well affect the rate of larval development. In particular, multiple studies (Botella et al. 1985; Borash et al. 1998; Sarangi et al. 2016) focused on the evolution of larval crowding in context of a tradeoff between tolerance to toxic nitrogenous waste and feeding rates, which is especially relevant in conditions of high animal density and limited food resources. It is possible that clustering animals reduce exposure to urea/ammonia by gaining access to deeper layers of food free of waste (which is unavailable for single diggers or uncoordinated foraging aggregations) and therefore, increase fitness without directly enhancing efficiency of food consumption or even at the cost of one. This phenomenon might be relevant for our system (where top layers of predigested food likely incorporate metabolic excretions of other larvae) and requires further examination.

In addition to the above, the influence of more complex and integrative factors on larval development in processed food and subsequent emergence of cooperative foraging is worth considering. As an example, it has been shown that gut microbiota in fruit flies is able to not only affect larval nutritional choices, feeding behavior (Venu et al. 2014; Leitão-Gonçalves et al. 2017; Qiao et al. 2019) and developmental rates (Shin et al. 2011), but also impacts kin recognition in adult flies (Lizé et al. 2013), which often is regarded as a source of social cooperation. Importantly, several studies suggest that kin recognition based on differences in gut microbiome community might also be manifested during larval stage and thus determine animals’ ability to engage in cooperative foraging as larvae (Khodaei and Long 2019) and other complex social interactions during adult stage (Carazo et al. 2015), both of which are associated with significant fitness benefits. This may be especially relevant in our model system, where food processing by the larvae most likely leads to profound changes in the local microbial environment. In support of this notion, a recent study by Klepsatel et al. (2018) found that rapid depletion of dietary yeast from the environment in case of high-density *Drosophila* larval populations may serve one of the main underlying reasons of larval crowding-associated fitness deficits. Therefore, future studies aimed to reveal connections between microbiome and cooperative foraging are required to shed more light on the evolution of social behaviors.

## Electronic supplementary material

Below is the link to the electronic supplementary material.

Supplementary file1 (TIF 1513 kb)

Supplementary file2 (TIF 1492 kb)
