# Supplementary figures and images for: Cooperative foraging during larval stage affects fitness in Drosophila
Source: J Comp Physiol A Neuroethol Sens Neural Behav Physiol. 2020 Jul 4;206(5):743–55. doi: 10.1007/s00359-020-01434-6 (PMC7392940; doi:10.1007/s00359-020-01434-6)

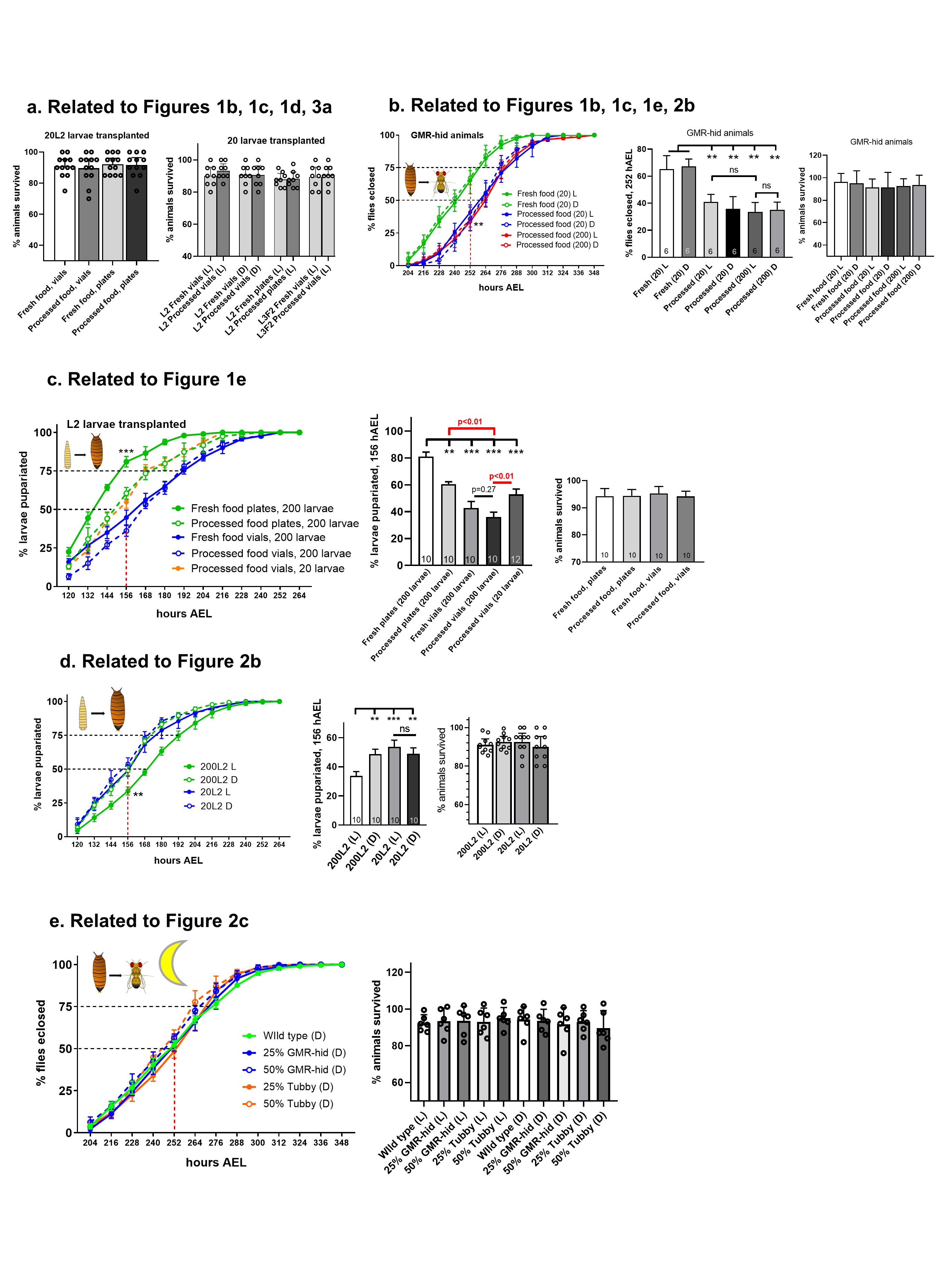

Supplement: Supplementary file 1 — Supplementary file1 (TIF 1513 kb) [file 359_2020_1434_MOESM1_ESM.tif]

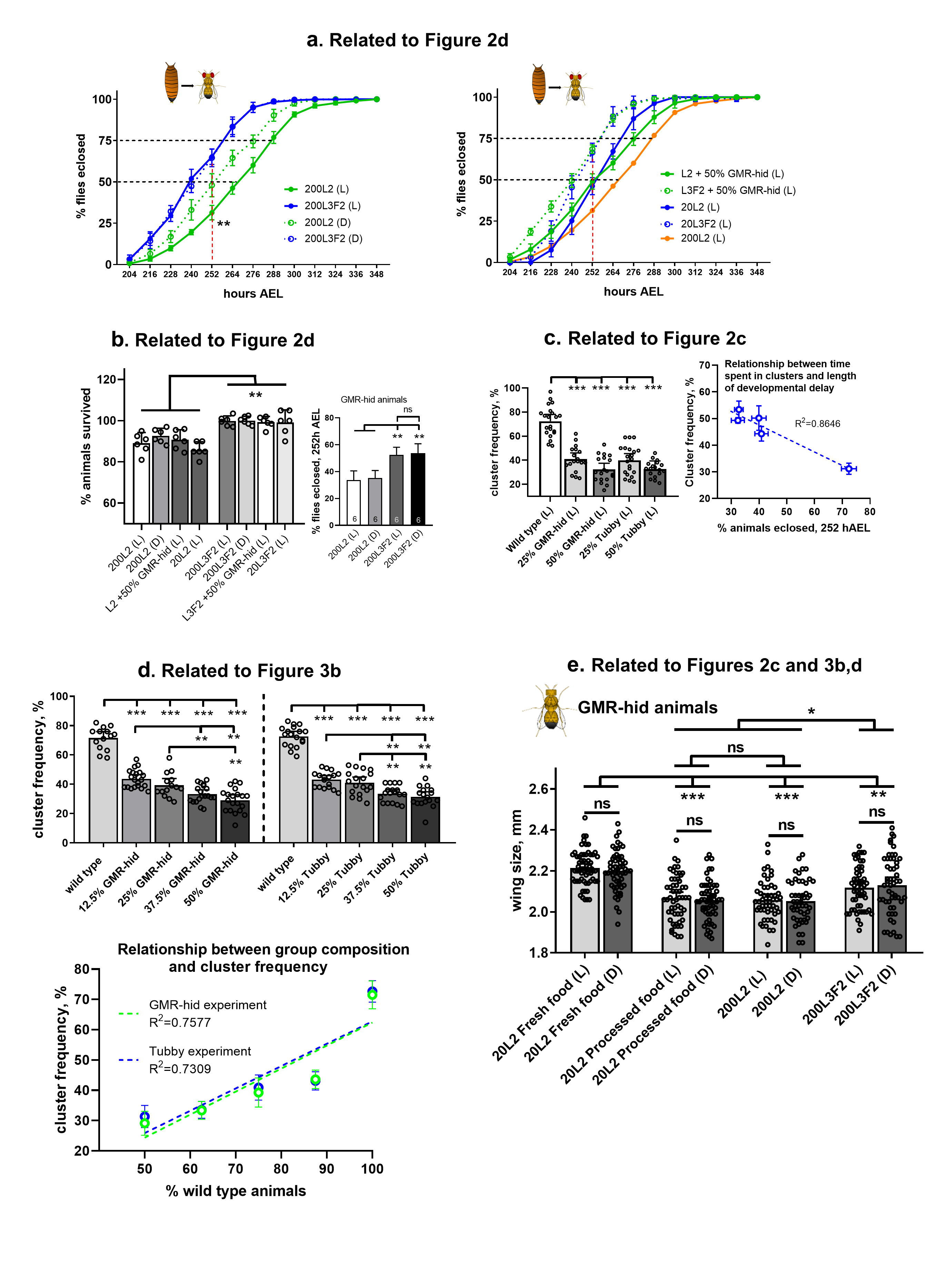

Supplement: Supplementary file 2 — Supplementary file2 (TIF 1492 kb) [file 359_2020_1434_MOESM2_ESM.tif]
